# Within species expressed genetic variability and gene expression response to different temperatures in the rotifer *Brachionus calyciflorus* sensu stricto

**DOI:** 10.1371/journal.pone.0223134

**Published:** 2019-09-30

**Authors:** Sofia Paraskevopoulou, Alice B. Dennis, Guntram Weithoff, Stefanie Hartmann, Ralph Tiedemann

**Affiliations:** 1 Unit of Evolutionary Biology/Systematic Zoology, Institute of Biochemistry and Biology, University of Potsdam, Potsdam, Germany; 2 Unit of Ecology and Ecosystem Modelling, Institute of Biochemistry and Biology, University of Potsdam, Potsdam, Germany; 3 Berlin-Brandenburg Institute of Advanced Biodiversity Research (BBIB), Berlin, Germany; 4 Unit of Evolutionary Adaptive Genomics, Institute of Biochemistry and Biology, University of Potsdam, Potsdam, Germany; National Cheng Kung University, TAIWAN

## Abstract

Genetic divergence is impacted by many factors, including phylogenetic history, gene flow, genetic drift, and divergent selection. Rotifers are an important component of aquatic ecosystems, and genetic variation is essential to their ongoing adaptive diversification and local adaptation. In addition to coding sequence divergence, variation in gene expression may relate to variable heat tolerance, and can impose ecological barriers within species. Temperature plays a significant role in aquatic ecosystems by affecting species abundance, spatio-temporal distribution, and habitat colonization. Recently described (formerly cryptic) species of the *Brachionus calyciflorus* complex exhibit different temperature tolerance both in natural and in laboratory studies, and show that *B*. *calyciflorus* sensu stricto (s.s.) is a thermotolerant species. Even within *B*. *calyciflorus s*.*s*., there is a tendency for further temperature specializations. Comparison of expressed genes allows us to assess the impact of stressors on both expression and sequence divergence among disparate populations within a single species. Here, we have used RNA-seq to explore expressed genetic diversity in *B*. *calyciflorus* s.s. in two mitochondrial DNA lineages with different phylogenetic histories and differences in thermotolerance. We identify a suite of candidate genes that may underlie local adaptation, with a particular focus on the response to sustained high or low temperatures. We do not find adaptive divergence in established candidate genes for thermal adaptation. Rather, we detect divergent selection among our two lineages in genes related to metabolism (lipid metabolism, metabolism of xenobiotics).

## Introduction

Within species genetic divergence can be influenced by multiple factors, including phylogenetic history, gene flow, genetic drift, and divergent selection [1]. The utilization of comparative transcriptomics allows us to assess the contribution of particular stressors to expression and sequence divergence among populations. This approach has been used to identify loci of ecological and evolutionary interest in a wide variety of taxa [2].

Rotifera is a highly diverse phylum that is comprised of more than 2000 species [3]. Understanding their adaptive diversification is fundamental because of their key role in energy transfer from primary producers to higher trophic levels in aquatic food webs [4]. Within Rotifera, cryptic speciation has been identified in at least 42 species, and has been related mainly to ecological factors [5]. In order to better understand the adaptive processes that may have led to such ecological speciation, knowledge of adaptive diversification among disparate populations within species and the underlying genetic variation is of essential importance. This is because local adaptation within species may constitute the starting point of further diversification and can ultimately lead to speciation [6–8].

Despite their potential for long-distance dispersal and high gene flow [9], rotifers exhibit profound within-species genetic differentiation [10–13]. These deep divisions within species can be promoted by local adaptation and explained via the Monopolization Hypothesis, according to which locally adapted populations have a competitive advantage over newly invading genotypes [14]. Zooplankton populations have shown signatures of local adaptation to several stressors. In evolution experiments with *Daphnia magna*, signatures of local adaptation were detected in genes related to anthropogenic stressors [7]. Campilo et al. [15–16] found signatures of divergent selection between populations in a common garden experiment in clones of *Brachionus plicatilis* from Spain, potentially reflecting adaptation to habitat differences (e.g., in the length of the growing season or the degree of habitat predictability). Recently, Franch-Gras et al. [17] found high within-population genetic variation in two life-history traits associated with diapause in nine populations of the rotifer *B*. *plicatilis* inhabiting saline lakes with varying degrees of environmental predictability.

Among abiotic factors, temperature is especially relevant for ectotherms. Temperature increases metabolic rates and affects all metabolic pathways, with potentially profound impacts on an organism’s survival and performance [18,19]. In aquatic ecosystems, temperature has been shown to impact species abundance, spatio-temporal distribution, and habitat colonization [20–22]. Expressed genetic diversity may underline the variation in heat tolerance observed within and between species. In rotifers, temperature impacts the occurrence of different cryptic species in both *B*. *plicatilis* and *B*. *calyciflorus* complexes [8, 22–24]. Both species complexes contain cyclical parthenogens, and their life history traits such as lifespan and reproduction are affected by temperature [25–27]. In a regional study in China, it was found that the species in the *B*. *calyciflorus* species complex, now known as *B*. *fermandoi* and *B*. *calyciflorus* sensu stricto (s.s.), have a tendency for specialization towards low and high temperatures, respectively [22]. A comparative laboratory study among these species also revealed significant differences in heat tolerance, with *B*. *calyciflorus* s.s. characterized as a thermotolerant species [24]. In the same study, albeit less pronounced, there was a tendency for further temperature specialization between clones within the more heat-tolerant *B*. *calyciflorus* s.s. with critical thermal maximum (CT_*max*_) between 42 °C and 60 °C [24]. Temperature has been found to differentially affect the growth rate and average life-span among clones from the same *B*. *calyciflorus* mtDNA evolutionary lineage [27].

The rapid development of high-throughput sequencing technologies and whole transcriptome profiling (RNA-seq) has enabled the deeper investigation of adaptive and functional variation in model and non-model species [28]. Several studies have investigated within and among species variation in transcriptional regulation of gene expression in zooplankton including daphnids and copepods, but such information is scarce in rotifers [29–31]. Gene expression studies in rotifers have focused primarily on identifying candidate genes relevant to the evolution of sex, aging and xenobiotics [32–34]. Thus far, expression changes in response to temperature changes have only been evaluated for candidate genes known for thermal response, such as heat-shock protein genes (*HSPs*) [35,36]. Furthermore, these studies have focused on the effect of acute heat stress, while information on gene regulation under prolonged heat exposure is missing. Three heat shock proteins have been suggested to be involved in thermal tolerance in rotifers: *HSP40*, *HSP60*, and *HSP70*. In particular, expression of the *HSP70* and *HSP40 gene* families were shown to impact heat shock survival in *B*. *manjavacas*, suggesting that there may be coordination among these in which *HSP40* works synergistically to regulate *HSP70*’s activity [35].

In the present study, we performed a temperature-specific RNA-seq experiment with sustained exposure to 14 °C and 26 °C in two phylogenetically and geographically disparate evolutionary lineages of the heat tolerant *B*. *calyciflorus* s.s.. Temperatures were selected to imitate conditions commonly found in nature towards their lower and upper thermal tolerance limits, hence focusing on adaptation to different ambient temperatures rather than thermal stress limits. We investigated both sequence divergence and gene expression among lineages within a single species. We identified putatively positive and negative selected genes that may reflect a different adaptive history of the respective lineages in nature and can be used as candidate genes to further investigate local adaptation. We did not find evidence of divergent selection between the clones in thermal stress related genes. Instead, we identified signatures of divergent selection in genes related to lipid metabolism and metabolism of xenobiotics. We further identified temperature-specific, differentially expressed genes that can be used as candidate genes to better understand temperature response and to identify environmental susceptibility with respect to temperature.

## Materials and methods

### Clonal origin and mtDNA phylogenetic history reconstruction

We selected two clones of *B*. *calyciflorus* s.s. from different continents with different phylogenetic history/genetic background to study potential adaptive variability and to unravel common temperature-dependent gene expression with different phylogenetic history/genetic background. The chosen clones were the strain IGB, originating from Germany (hereafter GER) and the strain Oneida, from Oneida Lake, USA (hereafter USA). Previous experiments showed a tendency for thermal tolerance variation among these clones (S1 Fig): estimated CT_*max*_ for GER clone was 43.18 °C while for USA 49.27 °C [24]. Both clones have been reared in the lab for at least 20 years at 20 °C under a 16:8 h light:dark photoperiod. Although both clones had the ability for meiotic reproduction at the time of collection (before 2000), this has been lost during the constant conditions of lab cultivation.

Species assignment of our clones was confirmed in a previous study using the ITS1 marker [24]. To further assign our clones to mitochondrial phylogenetic lineages, we utilized an established COI marker [24] and compared our clones to additional COI sequences assigned to *B*. *calyciflorus* s.s. species [11] which were downloaded from Gene bank (Table A in S1 Appendix). COI Sequences were aligned using ClustalX and a Bayesian phylogenetic tree was constructed by running BEAST 1.8.1 [37] for 30,000,000 generations. Genetic distance for the COI marker (i.e., average nucleotide diversity) between the two clones was estimated with MEGA v.5 [38] (S1 Supporting information).

### Clone cultivation and sample collection for the RNA-seq experiment

Temperature-specific experiments were performed at 14 °C and 26 °C with an exposure time of 30 days. Selected temperatures were within the tolerance range of the species [23], towards their upper and lower limits, respectively, which can be sustained for a prolonged period of time by both clones. Prolonged exposure was applied to mimic long-term exposure under these temperatures and to investigate potential divergent thermal adaptation among our clones. To acclimatize the clones to different temperatures, we increased or decreased the temperature 2 °C every 2 days until it reached the experimental temperature. Rotifers of both clones were then reared in 200ml glass flasks and exposed to constant temperature conditions at 26 °C or 14 °C. All rotifer cultures were reared in WC medium [39] under a 16:8 h light:dark photoperiod. A food combination of three algae species was provided every two days during the acclimatization and experimental period (*Monoraphidium minutum*; 10^6^ cells × ml^-1^, *Cryptomonas* spp: 5 × 10^4^ cells × ml^-1^, *Chlamydomonas reinhardii*; 5 × 10^5^ cells × ml^-1^).

Collection of rotifers took place during the light phase of cultivation and at the same time for all samples (two temperature conditions x two clones). Individuals of all stages were filtered through a 30μm sieve. The retained rotifers were re-suspended in WC medium in 50 ml-centrifuge tubes and centrifuged at 2,000 ×g for 10 minutes under the experimental temperatures (14 °C or 26 °C). Phytoplankton and other debris were pelletized at the bottom of the tube, while living rotifers remained suspended in the column. Without disturbing the pellet, rotifers were transferred by pouring into new 50 ml-centrifuge tubes containing 300ul of TRIzol^®^ LS reagent. Tubes were briefly vortexed and centrifuged again at 2,000 ×g, 4 °C for 1 minute. Pelletized rotifers were picked up and transferred into 2 ml-centrifuge tubes and centrifuged at 10,000 ×g for 1min. Any residual medium was carefully removed by pipetting, and 1ml of fresh TRIzol^®^LS reagent was added [40]. Samples were stored at -80 °C until RNA extraction. In total, four samples were collected, one sample per clone and temperature. Each sample was comprised of approximately 1,000 rotifer individuals.

### RNA extraction, library preparation, and sequencing

Samples in TRIzol^®^LS were homogenized using a plastic sterilized pestle and incubated overnight at room temperature. A total of 500μl of Chloroform was added to each sample and samples were centrifuged at 12,000 ×g for 15 minutes at 4°C to facilitate phase separation. We then transferred the colorless, upper aqueous phase into an RNeasy^®^ Mini Kit column (Qiagen, Germany) and proceeded to RNA precipitation and elution according to the manufacturer’s instruction. Total RNA concentration was estimated using a NanoDrop 1000 spectrophotometer (ThermoFischer Scientific, Germany). Quality of total RNA was examined using Agilent Bioanalyzer 2100 (Agilent Technologies, USA). RNA integrity (RIN) estimates were not applicable due to the presence of a “hidden break”, which led to a formation of only one strong 18S peak and a much reduced one for 28S rRNA [41].

For transcriptomic library preparation, mRNA was enriched from 3μg of total RNA with poly (A) capture using NEXTflex^™^ Poly (A) Beads, and strand-specific libraries were built using the NEXTflex^™^ Rapid Illumina Directional RNA-Seq Library Prep Kit (Bioo Scientific, USA) according to manufacturer’s instructions. Libraries were quantified using the Qubit dsDNA HS Assay Kit (Invitrogen, Germany). Quality control was performed using an Agilent Bioanalyzer 2100 (Agilent Technologies, USA). Libraries were sequenced for 150 bp, paired-end (PE) reads using an Illumina NextSeq 500 platform. Raw data presented here have been deposited in the NCBI Short Read Archive under the accessions numbers (SRA: SRR9040995-8).

### Intra-species variant calling and putatively positive and negative selected genes

Raw sequences were quality-checked using the FastQC v0.11.5 software [42]. Adapter sequences and low quality raw reads were trimmed using a 4 bp sliding window with a mean quality threshold of 15 and minimum read length of 36 bp with Trimmomatic v0.36 [43]. To call variants between the GER and USA clones, we used the SuperTranscripts pipeline, applied via Trinity v2.5.1 [44] separately for each clone (GER or USA). SuperTranscripts are created from all of the unique exonic sequences in a gene, in transcriptional order. This means that each gene is represented by a single sequence [45]. Although these SuperTranscripts do not necessarily represent any true biological molecule, they provide a good substitute for a reference genome [45].

To ensure that we only called SNPs that were fixed in each clone, we ran the entire variant-calling pipeline twice, using each of the SuperTranscripts assemblies as a reference. First, we assembled the raw reads (from both temperatures) that belong to GER and USA clones separately creating a complete transcriptome for each clone to further look for fixed variants between the clones. We produced two assemblies called “GER-assembly” and “USA-assembly”. To filter out contigs belonging to contaminants, either algae or bacteria, we created a “filtering contaminants pipeline”. First, we created a local contaminant database using sequences downloaded from NCBI which belong to potential algae contaminants species present in the culture medium: *Monoraphidium minutum*, *Chlamydomonas reinhardtii*, and *Cryptomonas* sp. Then, we downloaded from NCBI the available transcriptome assembly of *Brachionus calyciflorus* species with accession number GACL00000000.1 [32] and we created a second database of the reference transcriptome. Using a custom perl script, reads matched to any of the two databases. Only contigs with a best hit to the *B*. *calyciflorus* reference transcriptome were retained; reads matched to both databases with a bit score difference less than 100 were discarded. We also removed ribosomal RNA reads by performing a blastn search (ncbi-blast-2.6.0) [46] with an e-value cutoff of 1e^-10^ on a local database consisting of 18S and 28S sequences of *Brachionus* species downloaded from NCBI.

**Fig 1:**
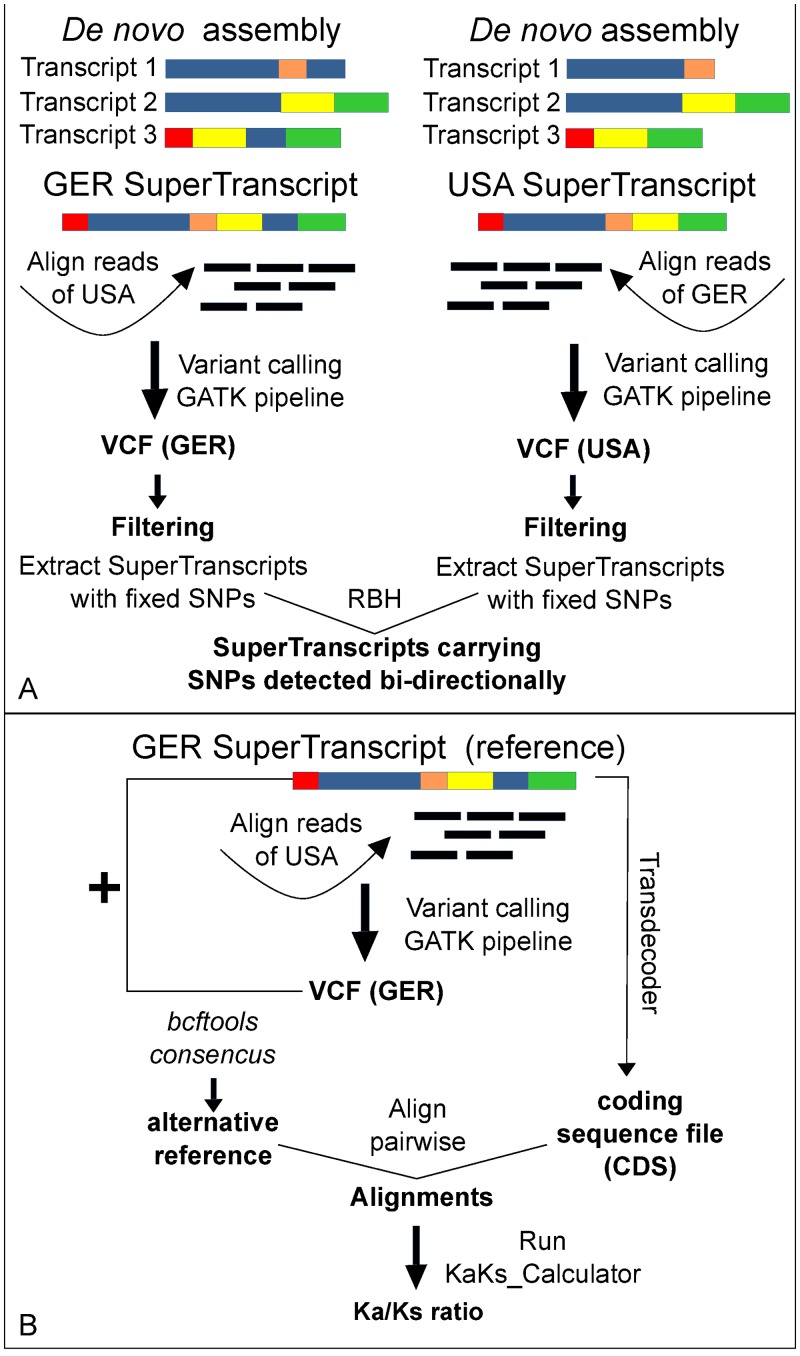
Bi-directional variant calling and identification of putatively positive selected genes as described in materials and methods. (A) Bi-directional variant calling pipeline between the two clones (GER, USA), (B) Ka/Ks ratio calculation pipeline.

Then, we used the “Trinity_gene_splice_modeler.py” with Trinity v2.5.1 [44] software to produce the SuperTranscript assemblies (GER-SuperTranscript, USA-SuperTranscript). To call variants, the “GER-SuperTranscript” assembly was used as a reference to align reads originating from the USA clone and vice versa (Fig 1A). We then used the “run_variant_calling.py” script from Trinity v2.5.1 [44] two times independently with the default parameters.

We applied further filters to a) remove INDELs using vcftools v0.1.14 [47] and b) retain only the SNPs fixed (i.e., homozygous) within a clone, using bcftools v1.6 [48]. From the assemblies of both clones, we extracted the SuperTranscripts containing fixed SNPs and we used a reciprocal best hit method (RBH) using BLASTN with a cutoff e-value of 1e^-50^ to identify putative orthologs present in both directions [49]. Only SuperTranscripts identified by RBH were further considered for our analyses. Since these two sets of genes were now identical subsets of SNPs identified by the bi-directional analysis, one of the assemblies (here, the “GER-SuperTranscript”) was further used as a reference.

To estimate heterozygosity, we aligned the reads originated from clone GER to the GER-SuperTranscript and reads originated from clone USA to the USA-SuperTranscript [45]. Only heterozygous SNPs, which are defined as those where two variants were detected within a clone and at least one read supporting the reference allele, were analyzed. Heterozygosity was calculated by dividing the number of sites carrying heterozygous polymorphisms to the total number of sites.

Open reading frames (ORFs) on the “GER-SuperTranscript” assembly were predicted by TransDecoder v.5.0.2 using the default parameters (https://github.com/TransDecoder/TransDecoder/wiki) and thus creating a coding sequence (CDS) file. We used *bcftools-consensus* to create an “alternative reference”, meaning a consensus sequence containing all alternative SNP variants relative to the reference file (bcftools v1.6). We used a custom script to a) trim both “reference” and “alternative reference” to the CDS positions predicted by TransDecoder, b) extract them in pairs and align them, and c) run KaKs_Calculator [50] using the MA model to estimate Ka/Ks ratio (Fig 1B). We used the ratio of non-synonymous substitutions per non-synonymous site (Ka) to the number of synonymous substitutions per synonymous site (Ks) to test for positive resp. negative selection. Sequences with a statistically significant (p < 0.05; evaluated with Fisher’s Exact test) Ka/Ks ratio above 1 (Ka/Ks > 1) were considered putatively under positive selection (i.e. divergent selection), while sequences with a statistically significant (p < 0.05) Ka/Ks ratio below 1 (Ka/Ks < 1) were considered putatively under purifying (i.e. negative) selection [51].

All genes carrying fixed SNPs were searched against the NCBI non-redundant (nr) database using the blastx algorithm with an e-value cutoff of 1e^-10^. Gene ontology (GO) assignment was performed with Blast2GO v14 [52]. We further used the online KEGG Automatic Server [53] for KEGG pathway analysis to categorize gene functions with an emphasis on those belonging to the same biochemical pathways. We tested both the putatively positive and negative selected genes for enriched KEGG pathways using a Fisher’s exact test implemented in R [54]. Putatively positive and negative selected genes that belonged to the enriched pathways were further tested for carrying mutations likely to alter protein function (ranked “deleterious”) on the Predict SNP server [55].

### Identification of temperature-specific differentially expressed genes in *B*. *calyciflorus* s.s.

To further analyze the species transcriptomic responses under different sustained temperatures, we *de novo* assembled all reads (both clones) in one combined assembly with Trinity v.2.5.1 software [44] using default parameters. After filtering for contamination, all unigenes were annotated against the KEGG database as described above for the independent assemblies.

To quantify expression, we used the Trinity v.2.5.1 software [44], incorporating the RSEM program [56]. To examine temperature-specific expression patterns in *B*. *calyciflorus* s.s., we compared expression between the two replicates (i.e., the two clones GER and USA) reared at 14 °C and the two reared at 26 °C, respectively, using EBseq [57]. EBSeq evaluates the posterior probability of differentially and non-differentially expressed entities (genes or isoforms) via empirical Bayesian methods. We considered as differentially expressed genes only those with a posterior probability equal to one (PPDE = 1). To reduce false positives, genes were considered distinctively expressed only, if they were significantly differentially expressed at a false discovery rate (FDR) below 0.05.

## Results

### Intra-specific genetic variation

Bayesian phylogenetic reconstruction assigned the USA and GER COI mtDNA haplotypes to different phylogenetic groups within *B*. *calyciflorus* s.s. with a posterior probability 0.88 (S1 Fig). Genetic distance between the USA and GER haplotypes for the COI marker was estimated to be approximately 6%. We further investigated the nuclear genetic divergence by calling variants between the GER and USA clones using SuperTranscripts in a bi-directional way. Trinity produced for the GER and USA assemblies 26,782 and 27,932 SuperTranscripts, respectively (Table B in S1 Appendix).

We used GER-SuperTranscript as a reference to align reads that originated from the USA clone in order to call variants, and vice versa. The number of variants, SNPs and fixed SNPs identified bi-directionally are presented in Table 1. Reciprocal best-hit identified 10,864 putative orthologs among the two clones of *B*. *calyciflorus* s.s., carrying 72,089 SNPs. Out of 10,864 SuperTranscripts, 8,573 had an open reading frame (ORFs) and at least one SNP within the coding region (CDS). These genes were considered for any further analyses. These 8,573 CDSs contained 52,102 SNPs of which 37,755 were synonymous and 14,347 were non-synonymous substitutions (Table 1).

**Table 1:** SNPs identified bi-directionally between GER and USA clones of *Brachionus calyciflorus* s.s. species.

	GER SuperTranscript used as reference	USA SuperTranscript used as reference
# variants (indels and SNPs)	123,058	134,070
# SNPs	110,762	123,592
# fixed SNPs	104,176	81,102
# SuperTranscripts with fixed SNPs	17,151	14,210
# SuperTranscripts with fixed SNPs (RBH*)	**10,864**	
# fixed SNPs in the SuperTranscripts (RBH*)	**72,089**	
# SuperTrancripts with ORFs and fixed SNPs in coding region (RBH*)	**8,573**	
# fixed SNPs in the coding region of the SuperTranscripts (RBH*)	**52,102**	
# Synonymous substitutions	**37,755**	
# Non-synonymous substitutions	**14,347**	

* RBH indicates that the intersect of the two datasets was determined by reciprocal-best blast hit

To estimate variant sites within the clones, we aligned the reads that originated from clone GER to the GER-SuperTranscript and reads that originated from USA clone to USA-SuperTranscript. In the case of the GER clone, we found 7,590 heterozygous SNPs belonging to 3,056 SuperTranscripts. In the case of USA clone, 39,157 SNPs belonging to 7,863 SuperTranscripts were found to be heterozygous. We calculated the observed heterozygosity by dividing the number of sites carrying polymorphisms with the total number of sites and we found that the USA clone is more heterozygous (0.138%) than the GER clone (0.026%) (Table C in S1 Appendix).

### Analysis and functional annotation of putatively positive and negative selected genes

**Fig 2:**
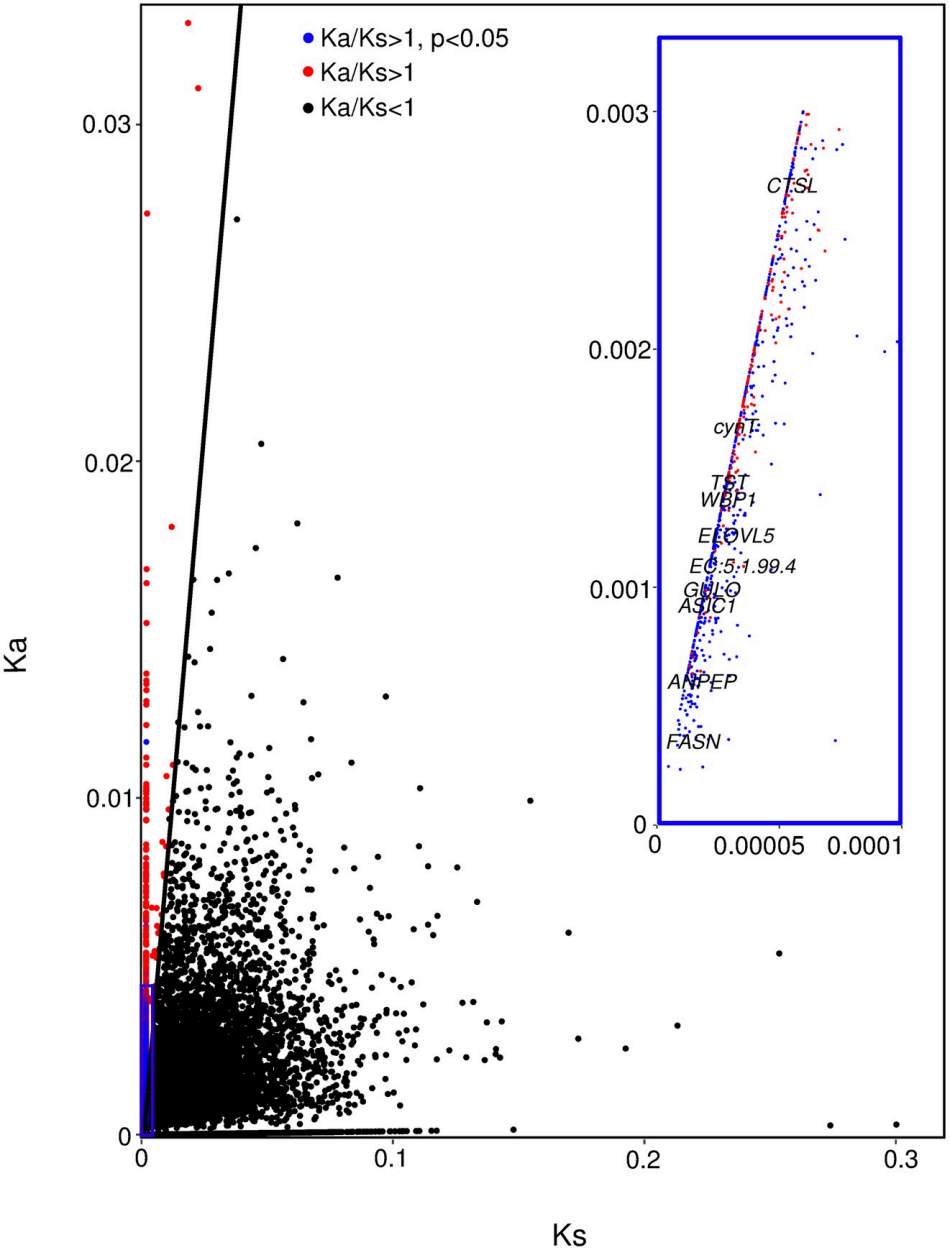
Distribution of Ka:Ks ratio. **Blue circles represent orthologous pairs with a statistical significant (p < 0.05) Ka/Ks > 1. Red circles represent all genes with a Ka/Ks > 1. Black line denotes the boundary between genes with a Ka/Ks > 1 and all other genes carrying SNPs (Ka/Ks < 1). Inset indicates genes belonging to the significantly enriched pathways and carrying potentially deleterious mutations**. *CTSL*; cathepsin, *cynT*; carbonic anhydrase, *TST*; thiosulfate/3-mercaptopyruvate sulfurtransferase, *WBP1*; oligosaccharyltransfe-rase complex subunit beta, E5.1.99.4; alpha-methylacyl-CoA racemase, *ELOVL5*: elongation of very long chain fatty acids protein 5, *GULO*; L-gulonolactone oxidase, *ASIC1*: acid-sensing ion channel 1, *ANPEP*; aminopeptidase N, *FASN*: fatty acid synthase, animal type.

**Fig 3:**
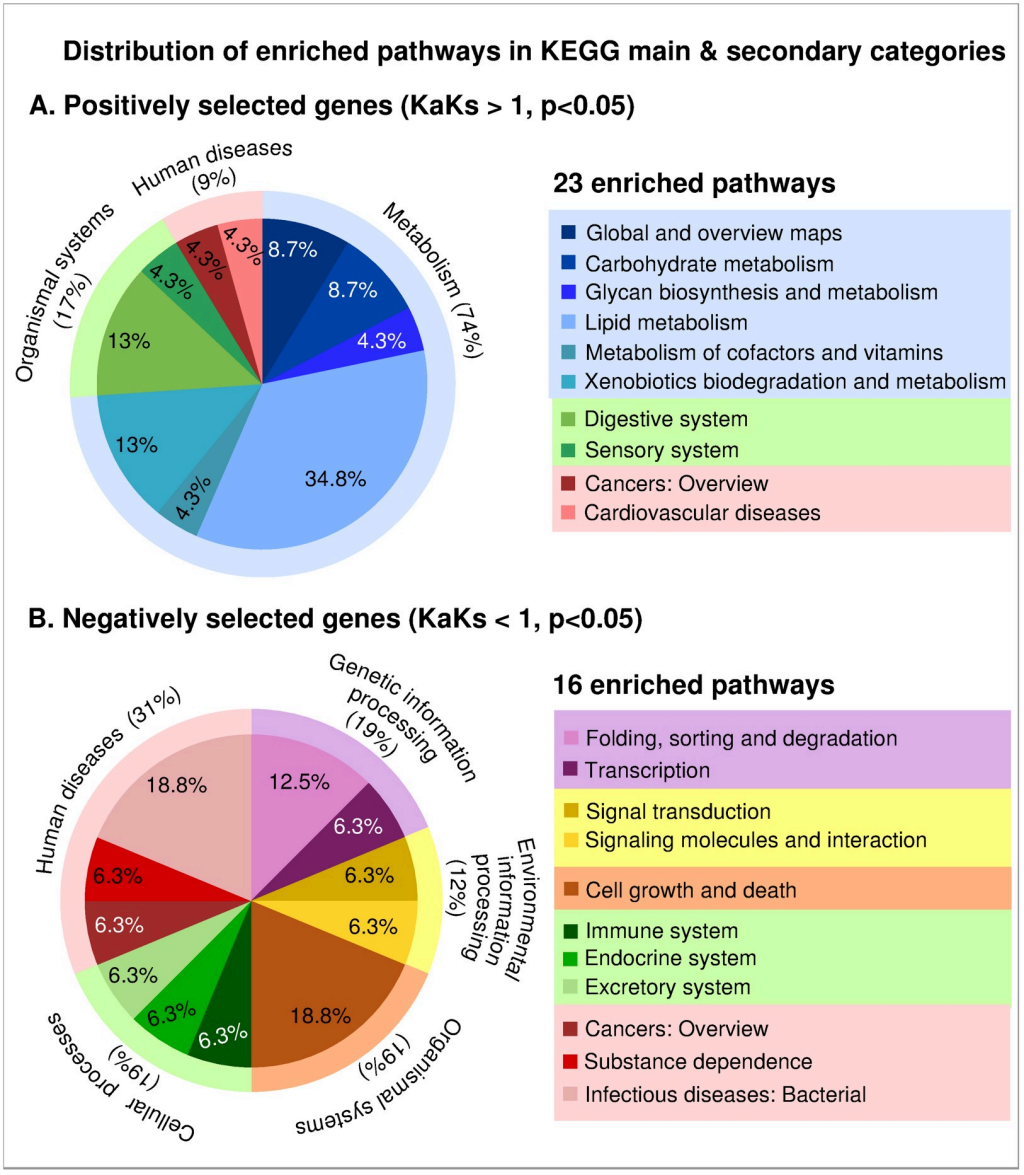
Distribution of pathways enriched in putatively A) positively selected genes, and B) negatively selected genes in main and secondary KEGG biological categories.

The estimation of Ka/Ks ratio for the 8,573 CDS pairs revealed 717 genes with Ka/Ks >1 (Fisher exact test: p<0.05), considered to be putatively under positive selection (i.e., divergent selection among the two evolutionary lineages analyzed here) and 6,434 genes with Ka/Ks < 1 (Fisher exact test: p < 0.05), considered to be putatively under purifying selection (Fig 2, Table D in S1 Appendix). We analyzed the 717 putatively positively selected genes with respect to their KEGG pathway annotation and found that 316 were assigned to a KEGG annotation term (KO) and belonged to 277 biochemical pathways (Tables E and F in S1 Appendix). Genes belonging to 23 different pathways were significantly enriched within the putatively positive selected genes, relative to all genes carrying SNPs (Fisher exact test: p<0.05) (Table F in S1 Appendix). The majority of these 23 pathways (17 pathways, 74%) belonged to the KEGG main biological category “Metabolism”. Within “Metabolism”, the categories “Lipid metabolism” (8 pathways), “Xenobiotics biodegradation and metabolism” (3 pathways) and “Carbohydrate metabolism” (2 pathways) were most represented (Fig 3A). Genes involved in all the above 23 pathways were further tested for mutations with likely implications on protein function (inferred as “deleterious”). In total, we found 9 genes carrying potential functionally relevant mutations in the USA clone which are represented on Fig 2 and Table 2. We also analyzed above genes with respect to their GO term annotations and we found that out of 717, four genes had a “*response to stimulus*” GO term (GO: 0050896), but none of these were related to heat stress (GO: 0009408) (Tables G and H in S1 Appendix).

**Table 2:** Putatively positively selected genes carrying mutations with potentially impacts on protein function.

Gene ID (GER-assembly)	Gene	KEGG Annot.	Ka/Ks	Length of amino acid chain	SNP position	Amino-acids in GER Allele*	Amino-acids in USA Allele*
DN11444_c1_g6	*CTSL*	K01365	50	144	22	S	W
DN11236_c3_g1	*ELOVL5*	K10244	50	316	76	P	A
DN12601_c3_g1	*WBP1*	K12670	50	268	64	N	Y
DN10357_c3_g2	*TST*	K01011	50	290	119	G	D
DN10310_c0_g1	*cynT*	K01673	50	264	204	Y	H
DN12142_c1_g1	*FASN*	K00665	33.4	981	973	V	G
DN5117_c0_g1	*GULO*	K00103	49.8	440	351	I	T
DN10824_c0_g1	*ANPEP*	K11140	39.2	580	575	Y	D
DN6362_c0_g1	*ASIC1*	K04829	50	459	203	L	S

* S: Serine, W: Thryptophan, P: Proline, A: Alanine, N: Asparagine, Y: Tyrosine, G: Glycine, D: Aspartic acid, H: Histidine, V: Valine, I: Isoleucine, T: Threonine, L: Leucine

We also analyzed the 6,434 putatively negatively selected genes with respect to their KEGG pathway annotation and found that 3,486 were assigned to a KEGG annotation term (KO) and belonged to 379 biochemical pathways (Tables I and J in S1 Appendix). Genes belonging to 16 different pathways were significantly enriched within the putatively negatively selected genes, compared to all genes carrying SNPs (Fisher exact test: p<0.05) (Table J in S1 Appendix). These 16 pathways belonged to “Human diseases” (5 pathways), “Genetic information processing” (3 pathways), Cellular processes” (3 pathways), “Organismal systems” (3 pathways), and “Environmental information processing” (2 pathways) KEGG main biological categories (Fig 3B). We also analyzed putatively negative selected genes with respect to their GO term annotations and we found that 64 genes had a “*response to stimulus*” GO term (GO: 0050896) among which genes encoding for heat shock protein 90 and 40, ras related proteins and DNA repair related proteins (Table H in S1 Appendix).

### Combined *de novo* assembly and gene functional annotation

For the differential expression analysis, both clones and temperature replicates were *de novo* assembled together. This combined *de novo* assembly with Trinity yielded 69,855 contigs after removing contaminants and ribosomal RNA. Clustering generated 37,785 unigenes with predicted ORFs in 16,489 (Table 3). A total of 6,098 unigenes were assigned to a KEGG annotation term (KO) and found to be involved in 394 biological pathways (Tables K and L in S1 Appendix).

**Table 3:** Summary statistics of the *de novo* combined assembly of the *Brachionus calyciflorus* s.s. transcriptome.

*B*. *calyciflorus* s.s. transcriptome assembly statistics	Combined assembly
# Raw reads (n)	218,952,060
# Trimmed and high quality raw reads assembled (n)	194,189,056
# assembled contigs (n)	90,011
# assembled contigs after contamination removal (n)	69,855
# assembled “unigenes” after removing contamination (n)	37,785
# predicted ORFs (n)	16,489
Average length (bp)	812
Median length (bp)	440
Total assembled bases (bp)	56,741,236
N50 (bp)	1,379
GC content for the entire assembly (%)	29.59

### Temperature-specific differentially expressed genes in *B*. *calyciflorus* s.s.

**Fig 4:**
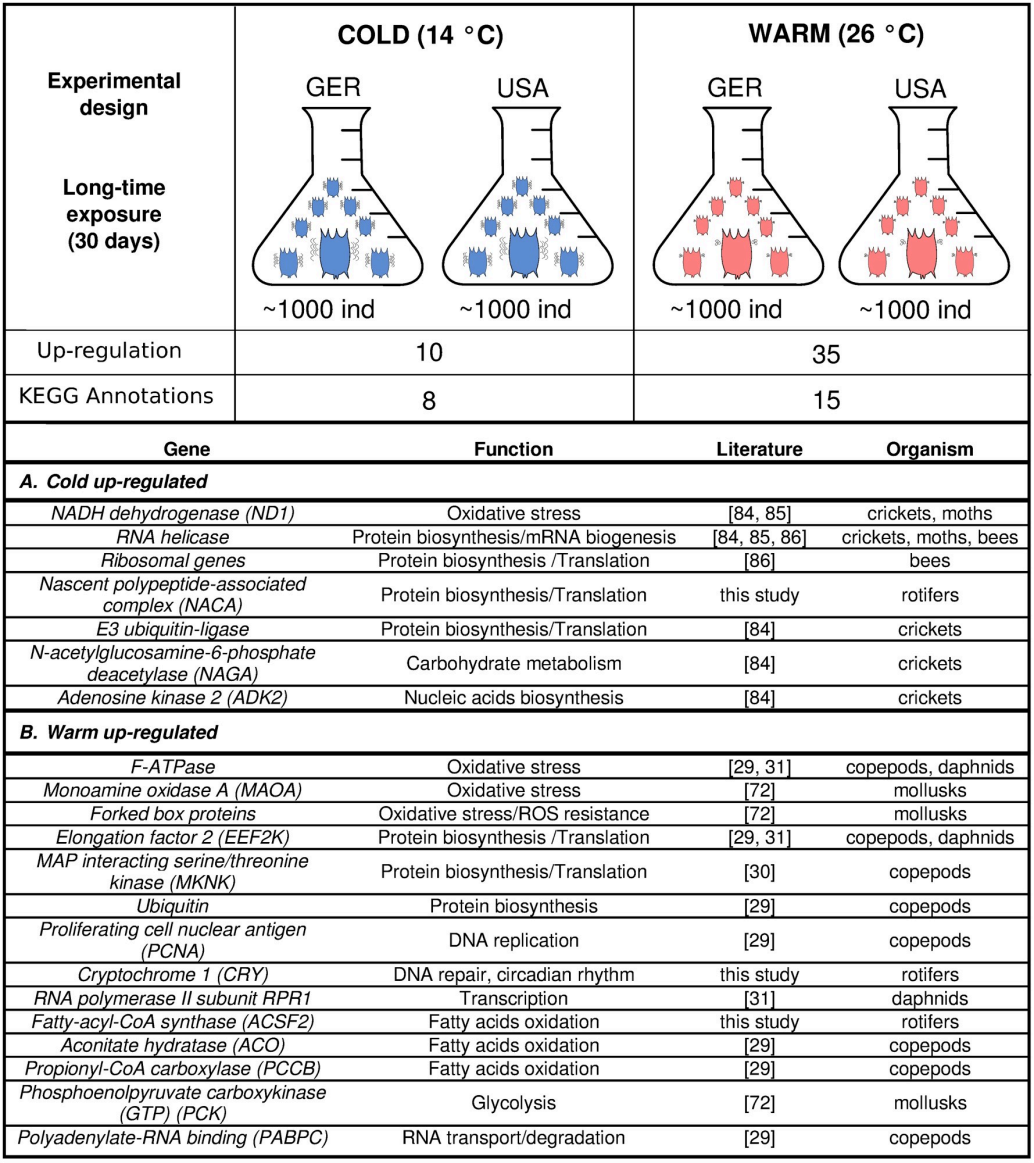
Experimental RNA-seq design and number of genes expressed differently after exposure to 14 °C and 26 °C along with their function. Genes previously reported in literature to be differentially expressed either under cold or heat stress are reported here along with a citation for the relevant study and the studied organism.

Between the two temperature regimes, we found 45 genes that were considered differentially expressed (PPDE = 1, FDR <0.05). Ten and 35 were up-regulated at 14°C and 26 °C, respectively (Fig 4, Table M in S1 Appendix). Among the two conditions, several genes relative to oxidative stress were differentially expressed (26 °C up-regulated: mitochondrial F–ATPase, probable flavin-containing monoamine oxidase A, Forked box proteins; 14 °C up-regulated: NADH dehydrogenase). In samples cultured at 26 °C we also found up-regulation of genes involved in DNA repair/replication (2 genes), fatty acid oxidation (3 genes), and protein biosynthesis (3 genes). In samples cultured at 14 °C, genes related to ribosomes (2 genes) and to translation and protein synthesis (4 genes) were also up-regulated. Most genes found in this study to be differentially expressed relative to temperature have been previously reported to be involved in temperature responses in various organisms (Fig 4).

## Discussion

### Intra-species genetic variation and putatively positive selected genes

In this study, we used RNA-seq to examine within species genetic variation with the potential to be adaptive. Applying a previously published evolutionary rate for mtCOI divergence of 2% per million years [58], the 6% difference found here between the USA and GER lineages indicates that these mitochondrial lineages may have split around 3 million years ago.

A total of 32% of all assembled genes were found to carry SNPs fixed for different bases in the two studied evolutionary lineages, indicating high among lineage genetic variation. We found a total of 6,434 and 717 genes putatively under purifying and divergent selection, respectively. Taking into account the mostly asexual reproduction and the similar treatment in cultivation, we argue that the signatures of positive selection we captured here may contain adaptations to environmental conditions in nature. However, divergence between the clones might have also occurred after these clones were isolated in the wild, especially during sexual reproduction and inbreeding in an early phase of lab cultivation. Under this hypothesis, adaptation to laboratory conditions via artificial selection might have evolved. After loss of sexuality, though, further adaptation to lab conditions may have slowed down, due to genetic uniformity and lack of recombination in these clonal lineages. In any case, our putatively positive selected genes exhibit faster diversifying evolution at non-synonymous sites than other genes and are hence worthy candidates to be further investigated regarding adaptive divergence.

The two lineages also differed in overall heterozygosity, with the USA clone being more heterozygous (0.138%) than the GER clone (0.026%). Rotifer species of the genus *Brachionus* are capable of clonal proliferation through parthenogenesis and sexual reproduction. Heterozygosity is often correlated with recombination rate, and recombination only occurs during the sexual phase of rotifers’ reproduction which can be triggered by unfavourable environmental conditions [59]. Whether the low observed heterozygosity in the GER clone reflects a lack of diversity in this lineage in nature cannot be determined here, as it might have resulted from heterozygosity loss under a combination of sexual reproduction and inbreeding in an early phase of lab cultivation.

We captured 64 genes under putative purifying selection that were annotated under the “*response to stimulus*” GO Term, including genes coding for heat shock proteins. This suggests that genes responsible for thermal adaptation are conserved between the two clones. In contrast, most of pathways enriched for putatively positive selected genes (i.e. genes under divergent selection) belonged to the main KEGG category “metabolism” (74%). Thus, adaptive divergence among our clones most likely takes place in genes related to metabolism. Within the category “Metabolism” there was a significant over-representation of pathways related to “Xenobiotics biodegradation and metabolism”, “Lipid metabolism”, and “Carbohydrate metabolism”.

In KEGG category “Xenobiotics biodegradation and metabolism”, we found signs of putative positive selection in two genes, glutathione S-tranferase (*GSTs*) and N-acetyl tranferase (*NATs*). Both genes play a significant role in detoxification and provide cellular protection against oxidative stress [60, 61]. This selective signal might reflect differences in the xenobiotics that different lineages experience in their natural environment or differences in their abilities to handle oxidative stress. Rotifers have been repeatedly suggested as potential model species for ecotoxicological studies due to their rapid responses to xenobiotic or toxic chemicals, and these genes may reflect adaptive divergence in such responses among our lineages [34].

We also inferred positive selection in genes related to “Lipid metabolism”. Among lipid metabolism genes, fatty acid synthase (*FASN*) and elongation of very long chain fatty acids protein 5 (*ELVOLV5*) were found to carry mutations potentially altering protein function (termed “deleterious” in the Predict SNP analysis). A deficiency in *FASN* is responsible for premature cell death and altered morphology in *Arabidopsis* [62]. *ELOVL5* and *ELOVL2* genes are involved in long-chain polyunsaturated fatty acid (PUFA) synthesis. Aquatic invertebrates modulate their PUFA biosynthesis through their diet and environmental factors including temperature and salinity [63]. Functional changes in genes belonging to Elovl family might be an indicator of adaptation to different diet composition in the natural environment. In rotifers, fatty acid composition of phospholipids is largely dependent on their food [64] and it has been shown that single clones respond differently to food compositions [65], giving further support to the idea that different lineages might be adapted to available food in their environment. Proportional changes of lipid composition in response to temperature changes are a major cellular response to adjust membrane fluidity. In aquatic invertebrates, alterations in temperature have been repeatedly connected to expression changes in genes related to lipid metabolism [63,66]. Several *ELOVL* genes have also been characterized for their adaptive function in *Brachionus koreanus*, and although their expression was downregulated after exposure to high salinity, the impact of functionally relevant mutations is still unknown [67,68]. To this extent, structural mutations in synthetase and elongase genes might be related to temperature adaptation, but further investigation is needed to make this conclusion.

In addition to KEGG categories related to metabolism, we found significant enrichment of putatively positive selected genes belonging to KEGG category “Sensory system”. Within this, the acid-sensing ion channel 1 (*ASIC1*) gene was found to carry functionally relevant mutations. In invertebrates, this gene is associated with membranes and ion channels. *ASIC* proteins are neuronal voltage-insensitive cationic channels that are activated by extracellular protons, and therefore respond to pH variation. The properties of *ASICs* proteins make these channels more suitable to sense dynamic pH fluctuations and increase cellular capacity to respond to prolonged or slow acidification [69]. Thus, significant variation in sensory related genes might be an indication of lineage-specific adaptations to local differences in absolute pH and/or the degree of pH fluctuations.

### Signatures of temperature-specific response

Among putatively selected genes, we found four genes that were annotated to the “*response to stimulus*” GO term, however none of these genes belonged to the “*response to heat*” GO term that nests within this, suggesting that there are not genetically-based differences in expressed genes specifically involved in thermal adaptation between the two mtDNA lineages. *Brachionus calyciflorus* s.s. species is a heat-tolerant species [24] and has been found under a range of temperature regimes, from 14 °C up to 35 °C [22], suggesting that it is capable of surviving a broad range of temperatures. As temperature affects all metabolic pathways and we found divergent selection in a number of metabolic pathways, the differences in thermotolerance among our two lineages may be associated with some of these pathways or lineage-specific differential gene expression in relevant pathways.

We investigated species-specific (i.e., consistent across lineages) transcriptomic responses to long-term exposure to high and low temperatures using our two investigated lineages as replicates. Prolonged exposure to high temperatures has been associated in other species with genes related to protein folding, oxidative stress, and immune function [31, 70–72]. Under long-term exposure at 26 °C, we found over-expression of oxidative stress related genes, including a Forkhead box (*F-BOX*) transcription factor, a mitochondrial *F–ATPase*, and a monoamine oxidase A (*MAOA*). High temperatures are associated with increased respiration, oxygen consumption and the production of toxic compounds that are called reactive oxygen species (*ROS*) [73]. *ROS* are highly reactive and can modify proteins, lipids, and nucleic acids, thereby contributing to the induction of cellular oxidative stress and cellular oxidative damage [73]. Activation of Forkhead transcription factors, such as forked box protein L, is a mechanism involved in reduction of *ROS* and promotes resistance to oxidative stress to improving survival, especially in cells that lack other mechanisms of such regulation [74]. Also up-regulated after prolonged exposure at 26 °C, mitochondrial *F-ATPase* is a key enzyme in metabolism and drives the synthesis of ATP. The same gene was differentially expressed in the copepod *Calanus finmarchicus* under long-term heat-stress [29]. Due to the complex and energy-demanding processes involved in the elevation of stress, a constant supply of ATP is required to restore cell homeostasis during heat stress, and this could explain the up-regulation of ATPase genes in samples exposed at 26 °C that were observed here across *B*. *calyciflorus* s.s. lineages. Over-expression of *F-ATPase* relevant proteins has also been observed to increase resistance to salts, drought, and oxidative stresses in less complex organisms such as yeast, suggesting that induction of the *F-ATPase* plays a role in stress tolerance [74]. Also up-regulated in our samples exposed at 26 °C, monoamine oxidase proteins are mitochondrial proteins that catalyze the oxidative deamination of amines, such as dopamine and norepinephrine. In mitochondria, acute heat-stress induces an increase in substrate oxidation and electron transport chain activity, while chronic heat stress can lead to shrinking of metabolic oxidative capacity [75].

We further identified up-regulation of genes involved in DNA repair/replication, fatty acid oxidation, and protein biosynthesis. Among genes associated with DNA repair/replication we identified up-regulation of proliferating cell nuclear antigen (*PCNA*). *PCNA* is involved in DNA synthesis during replication, DNA repair, and cell cycle control [76]. Under stress, the DNA synthesis process of gap-filling and nucleotide excision repair system is forced to stand-by until base excision repair system is completed. Such a delay of gap-filling of nucleotide excision repair system might cause a modest increase in cell death, which could be suppressed by over-expression of *PCNA* [76]. *PCNA was* also found to be over-expressed under long-term heat stress in the copepod *C*. *finmarchicus* [29]. Thus, it is possible in our case that cells may increase expression of *PCNA* to overcome cell death caused by prolonged exposure at 26 °C.

One important gene, found up-regulated in samples exposed at 26 °C and involved in AMP signaling, is the eukaryotic elongation factor-2 kinase (*EEF2K*). *EEF2K* inhibits eukaryotic elongation factor 2 and slows the elongation stage of protein synthesis, which normally consumes a great deal of energy and amino acids [77]. Up-regulation of *EEF2K* has also been observed after ribosomal stress, a well-known cellular response to abiotic stressors [78]. Another significant gene found to be up-regulated after sustained exposure at 26 °C was MAP kinase-interacting serine threonine (*MKNK*), which is involved in MAP signaling pathway and causes inactivation of eukaryotic initiation factor 4E (eIF4E), subsequently inhibiting translation [79]. Protein synthesis inhibition is an immediate response during stress to switch the composition of protein pool in order to adapt to new environments [77]. *MKNK* was also found to be up-regulated under acute heat stress in copepods [30]. In addition, we captured up-regulation of genes involved in activation of fatty acids oxidation, Propionyl-CoA carboxyla (*PCCB)*, and aconinate hydractase (*ACO*). These genes were also up-regulated under prolonged heat stress in copepods [29], indicating that this might be a consistent pattern in zooplankton species.

Prolonged exposure to low temperatures also triggered the over-representation of oxidative stress-related genes. We found over-representation (7x-fold change) of the gene NADH dehydrogenase (*ND1*), an important component of *ROS* production [80], indicating possible high oxidative stress after sustained exposure at 14 °C. In samples exposed to 14 °C, we also captured up-regulation of several genes related to ribosomes. Most of these genes are involved in functions such as translation and protein synthesis, and these included ribosomal structural genes (*60S-L26*, *60S-L21*) and a ribosome-associated nascent polypeptide-associated complex (*NACA*) gene [81], which is involved in multiple translational processes, including protein transport into the mitochondria and protein folding [81]. Up-regulation of ribosomal proteins in response to sustained stress has been found in the Pacific oyster, suggesting an effort to increase translation capacity or protect ribosomal function through the addition or replacement of ribosomal proteins [70]. Up-regulation of ribosomal related genes was previously found in *Daphnia* as a response to stress induced by predation [82] and in silkworms as a response to stress induced by fluoride [83]. This suggests that ribosomal gene products are important for metabolic adjustments during stress in general. Furthermore, nucleotide metabolism was altered under prolonged exposure to 14 °C, indicated by the increased accumulation of adenosine kinase 2 (*ADK2*). *ADK2* plays an important role in energy homeostasis and cellular responses to biotic and abiotic stress and has been reported as differentially expressed under low temperatures in crickets [84].

Interestingly, for several of the genes discussed above that are up-regulated in *B*. *calyciflorus* s.s. clones after prolonged exposure to 14 °C (*ND1*, ADK2, and ribosome-related genes), studies in crickets, bees, and moths have found down-regulation under cold conditions [84–86]. Similarly, opposing patterns of transcriptomic regulation have been observed in comparisons of heat tolerant and heat sensitive mollusk species [72]. As of now, we cannot explain these opposing patterns of expression relative to other organisms under thermal stress. However, we suggest that this shows that environmental temperature impacts expression of these genes across a range of invertebrate taxa.

## Conclusions

In this study we identified synonymous and non-synonymous SNPs between two substantially diverged mtDNA lineages of the species *B*. *calyciflorus* s.s., indicating a high level of within species expressed genetic variation. Among the variable genes, we identified candidates with signatures of positive selection, many of which have ecological relevance and could be used in future work studying adaptation. We showed that between the two clones, genes directly related to thermal adaptation are most likely under purifying selection. Finally, we identified temperature-specific differentially expressed genes which can be used to further test susceptibility of *Brachionus* to environmental stress.

## Supporting information

S1 Appendix Supporting data of putatively selected genes, differential expressed genes, GO term and KEGG annotations. (XLSX)

S1 Fig Thermal Death Time curves (TDT) for the two clones (GER, USA), showing the estimated CT_*max*_ value for each clone. (PNG)

S2 Fig Phylogenetic history reconstruction for the COI mtDNA marker within the species *B*. *calyciflorus* s.s. (PNG)

S1 Supporting information Additional information on COI mtDNA phylogenetic reconstruction. (DOCX)
