# Supplementary figures and images for: Within species expressed genetic variability and gene expression response to different temperatures in the rotifer Brachionus calyciflorus sensu stricto
Source: PLoS One. 2019 Sep 30;14(9):e0223134. doi: 10.1371/journal.pone.0223134 (PMC6768451; doi:10.1371/journal.pone.0223134)

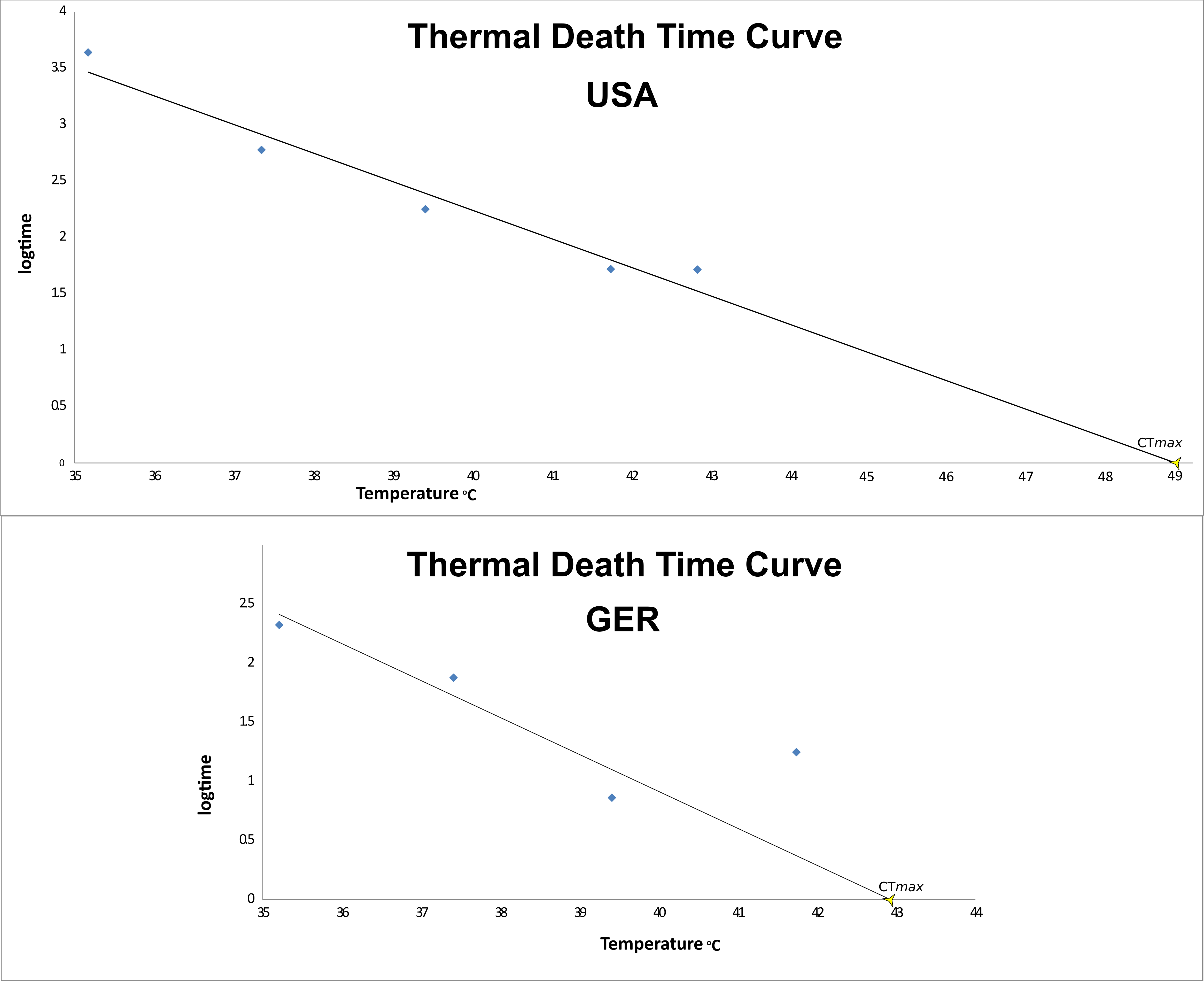

Supplement: S1 Fig — (PNG) [file pone.0223134.s002.png]

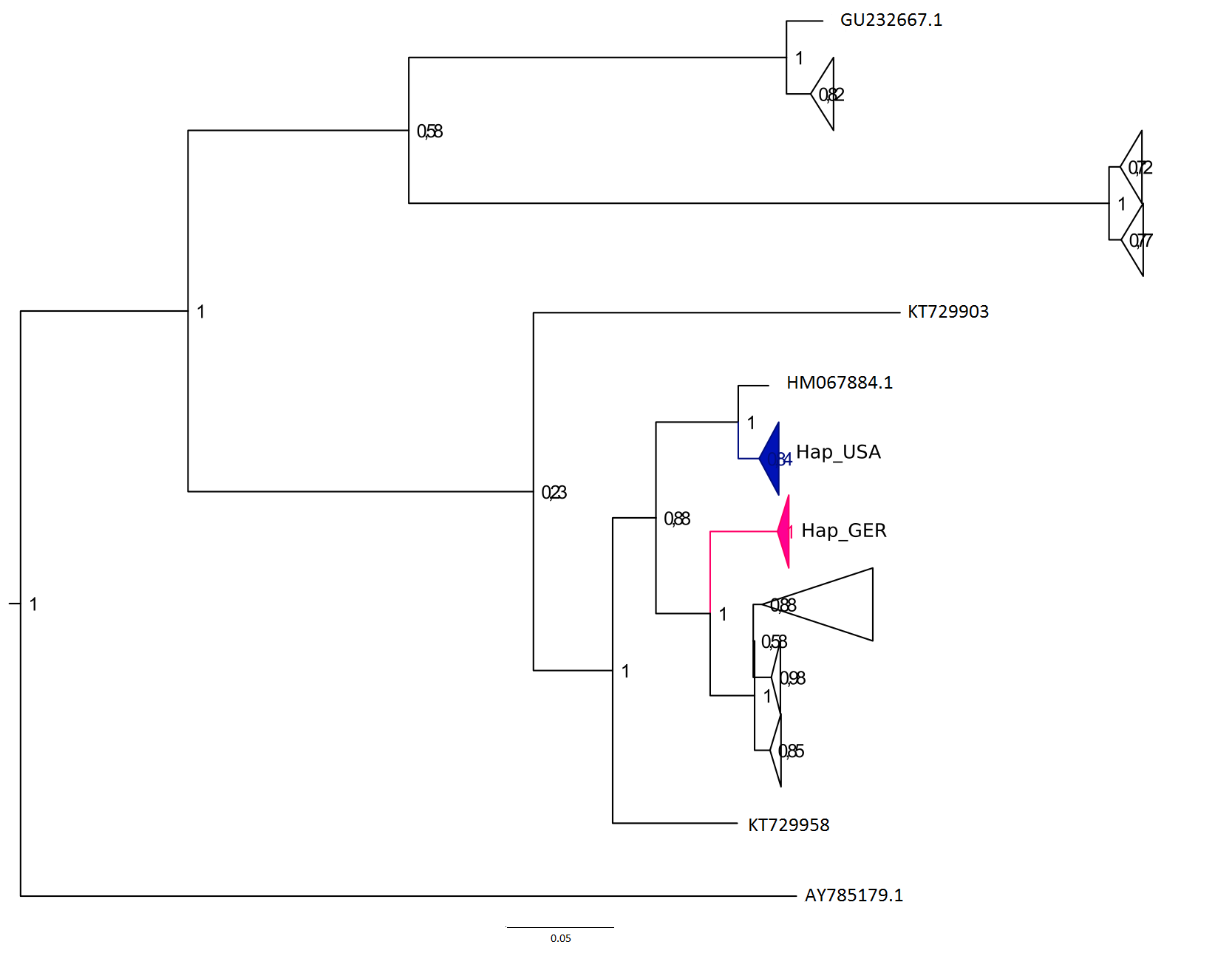

Supplement: S2 Fig — (PNG) [file pone.0223134.s003.png]
