# Supplementary material for: Within species expressed genetic variability and gene expression response to different temperatures in the rotifer Brachionus calyciflorus sensu stricto
Source: PLoS One. 2019 Sep 30;14(9):e0223134. doi: 10.1371/journal.pone.0223134 (PMC6768451; doi:10.1371/journal.pone.0223134)
Supplement: S1 Supporting information — (DOCX) [file pone.0223134.s004.docx]

**DNA extraction and PCR amplification conditions**

Both ITS1 (nuDNA) and COI (mtDNA) sequences from both GER and USA clones were obtained during previous work (Paraskevopoulou et al., 2018). The nuITS1 phylogenetic tree provided in Paraskevopoulou et al., 2018 was used for species assignation. To reconstruct the mDNA phylogentic history of *B. calyciflorus* we downloaded from the Gene Bank all available halpotypes for the species and we added the two haplotypes of our clones. Then we run a phylogenetic analysis using BEAST 1.8.1 (Drummond & Rambaut, 2007) for 30,000,000 generations with a sampling frequency of 3000 generations under a lognormal relaxed (uncorrelated) clock, with a constant-size coalescent tree prior (Papakostas et al., 2016). Following the Bayesian Information Criterion (BIC), the GTR+G and evolutionary models were used for nuITS1 and mtDNA markers respectively (Posada, 2008). The consensus tree was calculated after discarding the first 20% of the trees as burn-in.

**References**

Drummond AJ, Rambaut A. 2007 BEAST: Bayesian evolutionary analysis by sampling trees. BMC Evol. Biol. 7, 214. doi:10.1186/1471-2148-7-214

Papakostas S et al. 2016a. Integrative Taxonomy Recognizes Evolutionary Units Despite Widespread Mitonuclear Discordance: Evidence from a Rotifer Cryptic Species Complex. Syst. Biol. 65, 508–24. (doi:10.1093/sysbio/syw016)

Paraskevopoulou S, Tiedemann R, Weithoff G. Differential response to heat stress among evolutionary lineages of an aquatic invertebrate species complex. Biol Lett. 2018; 14: 20180498. doi: 10.1098/rsbl.2018.0498

Posada D. 2008. jModelTest: Phylogenetic model averaging jModelTest: phylogenetic model averaging. Mol. Biol. Evol. 25, 1253–1256. (doi:10.1093/molbev/msn083)
